# In Vitro and In Vivo Effects of SerpinA1 on the Modulation of Transthyretin Proteolysis

**DOI:** 10.3390/ijms22179488

**Published:** 2021-08-31

**Authors:** Filipa Bezerra, Christoph Niemietz, Hartmut H. J. Schmidt, Andree Zibert, Shuling Guo, Brett P. Monia, Paula Gonçalves, Maria João Saraiva, Maria Rosário Almeida

**Affiliations:** 1 Molecular Neurobiology Group, i3S-Instituto de Investigação e Inovação em Saúde, IBMC-Instituto de Biologia Molecular e Celular, Universidade do Porto, 4200-135 Porto, Portugal; carla.bezerra@ibmc.up.pt (F.B.); b12458@med.uminho.pt (P.G.); mjsaraiv@ibmc.up.pt (M.J.S.); 2 Departamento de Biologia Molecular, ICBAS-Instituto de Ciências Biomédicas Abel Salazar, Universidade do Porto, 4050-313 Porto, Portugal; 3 Medizinische Klinik B, Universitätsklinikum Münster, 48149 Münster, Germany; Christoph.Niemietz@taconic.com (C.N.); hepar@ume.de (H.H.J.S.); Andree.Zibert@ukmuenster.de (A.Z.); 4 Ionis Pharmaceuticals, Carlsbad, CA 92010, USA; sguo@ionisph.com (S.G.); bmonia@ionisph.com (B.P.M.)

**Keywords:** transthyretin, SerpinA1, ATTR amyloidosis, TTR proteolysis, plasmin

## Abstract

Transthyretin (TTR) proteolysis has been recognized as a complementary mechanism contributing to transthyretin-related amyloidosis (ATTR amyloidosis). Accordingly, amyloid deposits can be composed mainly of full-length TTR or contain a mixture of both cleaved and full-length TTR, particularly in the heart. The fragmentation pattern at Lys48 suggests the involvement of a serine protease, such as plasmin. The most common TTR variant, TTR V30M, is susceptible to plasmin-mediated proteolysis, and the presence of TTR fragments facilitates TTR amyloidogenesis. Recent studies revealed that the serine protease inhibitor, SerpinA1, was differentially expressed in hepatocyte-like cells (HLCs) from ATTR patients. In this work, we evaluated the effects of SerpinA1 on in vitro and in vivo modulation of TTR V30M proteolysis, aggregation, and deposition. We found that plasmin-mediated TTR proteolysis and aggregation are partially inhibited by SerpinA1. Furthermore, in vivo downregulation of SerpinA1 increased TTR levels in mice plasma and deposition in the cardiac tissue of older animals. The presence of TTR fragments was observed in the heart of young and old mice but not in other tissues following SerpinA1 knockdown. Increased proteolytic activity, particularly plasmin activity, was detected in mice plasmas. Overall, our results indicate that SerpinA1 modulates TTR proteolysis and aggregation in vitro and in vivo.

## 1. Introduction

Transthyretin-related amyloidoses (ATTR amyloidosis) are characterized by extracellular deposition of insoluble TTR amyloid fibrils in several tissues, being polyneuropathy and cardiomyopathy the major clinical manifestations, as reviewed in [1,2]. There are two types of ATTR amyloidoses: hereditary amyloidosis (ATTRm) and wild-type ATTR amyloidosis (ATTRwt) [3]. ATTRm occurs through single-residue substitutions in TTR, mainly producing less stable variants [4,5,6], whereas ATTRwt is an age-related disorder, affecting 20–25% of the population over 80 years, with predominant cardiac phenotype, characterized by wild-type (WT) TTR amyloid deposits [7,8].

Despite it is widely accepted that tetramer destabilization is a rate-limiting step for the development of amyloid fibrils [9,10,11,12], TTR proteolysis has been increasingly recognized as another mechanism driving TTR amyloid formation. Several studies reported the existence of different TTR amyloid deposits in a range of tissues. Thus, amyloid deposits might be composed mainly of full-length TTR (type-B fibrils) or by a mixture of both cleaved and full-length TTR (type-A fibrils) [13,14,15,16]. Type-A fibrils occur in several tissues, particularly in the heart and are related to the development of restrictive cardiomyopathy after liver transplantation, leading to poor clinical outcomes in ATTR V30M patients [17]. The protease responsible for TTR cleavage has not yet been identified. However, the specific fragmentation at Lys48 in the TTR polypeptide suggests that it could be a trypsin-like serine protease [18]. In vitro experiments using recombinant trypsin indicate that several amyloidogenic TTR variants are susceptible to trypsin-mediated proteolysis [19,20,21]. The process of cleavage and release of the 49–127 TTR fragment, the most frequent fragment detected in fibrils, is faster for the highly amyloidogenic variant, TTR S52P, being the 49–127 C-terminal fragment rapidly incorporated into amyloid fibrils [19,20].

Additionally, in silico studies pointed out plasmin as a plausible pathophysiological candidate protease involved in the process of TTR amyloid formation [22]. Furthermore, the ubiquitous distribution of plasmin, its structural similarities to trypsin [22], and the reported activation of plasminogen activation system (PAS) in other amyloid-related disorders, such as Alzheimer’s disease [23] and immunoglobulin light chain (AL) amyloidosis [24,25,26] indicate that this protease could have a key role in TTR amyloidogenesis. Indeed, the 49–127 C-terminal TTR fragment was also found in in vitro plasmin-digested TTR S52P samples, suggesting that TTR-digested samples are more prone to aggregate than the non-digested ones [22].

On the other hand, serine protease inhibitors (Serpins), particularly SerpinA1, have also been related to pathological proteinopathies, such as Alzheimer’s disease [27,28,29]. We hypothesized that SerpinA1 may act as a modulator of TTR proteolysis/fibrillogenesis. SerpinA1 belongs to the clade A serpins, which are classified as antitrypsin-like [30]. SerpinA1 and also SerpinA3 were found to be differentially expressed in ATTRm patients compared to healthy controls [31,32]. The SerpinA1 mRNA was found to be differentially expressed in hepatocyte-like cells (HLCs) from ATTR patients compared to healthy controls and, a high inverse correlation between *SerpinA1* and *TTR* genes was also observed. Upon TTR knockdown in HLCs the correlation was abolished [33]. Recently, it was demonstrated that SerpinA1 knockdown modulates TTR expression in both cellular and animal models, performing an important role in the development of ATTR amyloidosis [34].

In this study, we further explored the role of SerpinA1 on the in vitro and in vivo modulation of plasmin-mediated TTR proteolysis and how this modulation may impact TTR amyloidogenesis to contribute to the development of more targeted therapies for the treatment of ATTR amyloidosis.

## 2. Results

### 2.1. SerpinA1 Inhibits In Vitro Plasmin-Mediated Proteolysis of Transthyretin V30M

**Figure 1:**
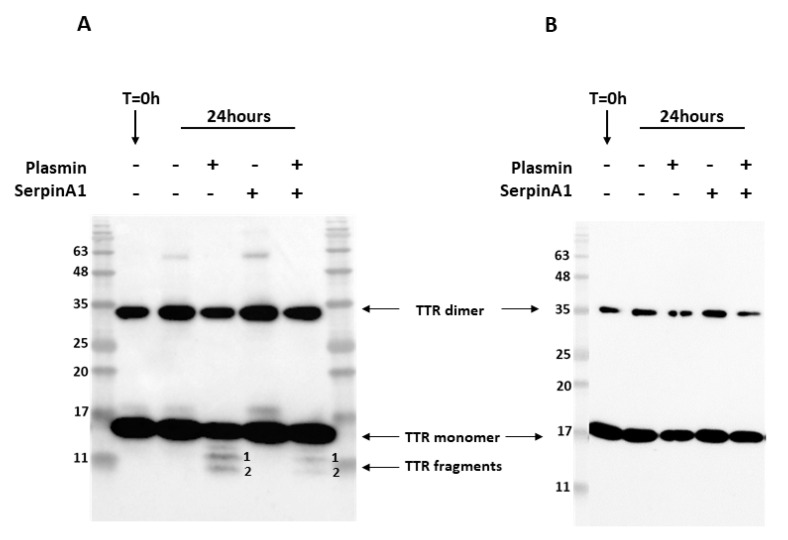
Plasmin cleaves transthyretin V30M, and its activity is inhibited by SerpinA1. Representative images of Western blotting analysis of the three independent in vitro experiments. Western blot was performed using two different antibodies targeting human TTR, mouse anti-TTR mutant (Y78F), clone AD7 (**A**), and rabbit anti-transthyretin (**B**). Both antibodies detected dimeric and monomeric TTR forms. However, only the mouse anti-TTR mutant (Y78F) clone AD7 detects TTR fragments (**A**).

Previous studies revealed that similarly to TTR S52P, recombinant TTR V30M was also susceptible to plasmin-mediated proteolysis [22]. In addition, amyloid deposits extracted from cardiac and adipose tissue specimens from ATTR V30M patients were composed of a mixture of both cleaved and full-length TTR [13,14,15]. Thus, we performed in vitro proteolysis experiments to evaluate whether SerpinA1 performs a role on the inhibition of plasmin-mediated TTR V30M proteolysis. Recombinant TTR V30M was incubated with plasmin at 37 °C for 24 h, and the resulting mixture was analyzed by SDS-PAGE and Western blot. Besides the dimeric (~35 kDa) and monomeric (~17 kDa) TTR V30M forms, Western blotting analysis revealed the presence of two different TTR fragments, in contrast to non-digested samples (Figure 1A and Supplementary data Figure S1). These fragments were detected with the commercially available antibody produced and characterized in our lab [35], anti-TTR mutant (Y78F), clone AD7 (Figure 1A) but not with rabbit polyclonal anti-TTR from DAKO (Figure 1B).

N-terminal sequencing analysis of the TTR fragments firstly observed in Western blotting analysis indicates that band 1 corresponds to a peptide starting at position 49 and band 2 to a peptide starting at the first amino acid of TTR polypeptide chain (Table 1). Furthermore, the bands corresponding to TTR fragments were excised from SDS-PAGE gels and further analyzed by mass spectrometry (MS) analysis after trypsin digestion. These MS experiments revealed that band 1 was composed of the peptides with mass corresponding to the amino acids 81–103, 104–127, 105–126, and 105–127 (Table S1), which along with N-terminal sequencing data, indicated that band 1 should correspond to the TTR fragment 49–127 (Table 1). Band 2 was composed by the peptides with mass corresponding to the amino acids 1–15, 22–34, 35–48, and 36–48 (Table S1). Together with N-terminal sequencing data, our results indicate that band 2 was the TTR fragment 1–48 (Table 1). Intriguingly, the band containing 1–48 N-terminal TTR fragment also revealed the presence of a C-terminal peptide comprising the amino acids 105–127. Since the number of peptide-spectrum matches (PSMs) identified for that peptide group was only two, which was too low compared to the N-terminal peptides, this might indicate that this C-terminal peptide 105–127 was a contaminant.

Following, we investigated the role of the serine protease inhibitor, SerpinA1, as a modulator of plasmin-mediated TTR proteolysis. We found that TTR proteolysis was partially inhibited in the presence of SerpinA1 (Figure 1A and Figure S1). No TTR fragments were observed neither before the assay nor in the absence of plasmin, excluding the influence of TTR auto-proteolysis or degradation. In opposition to TTR V30M, TTR WT was not susceptible to plasmin-mediated proteolysis under the same conditions as presented in supplementary data (Figure S2).

### 2.2. SerpinA1 Inhibits In Vitro Transthyretin V30M Aggregation upon Plasmin-Mediated Proteolysis

**Figure 2:**
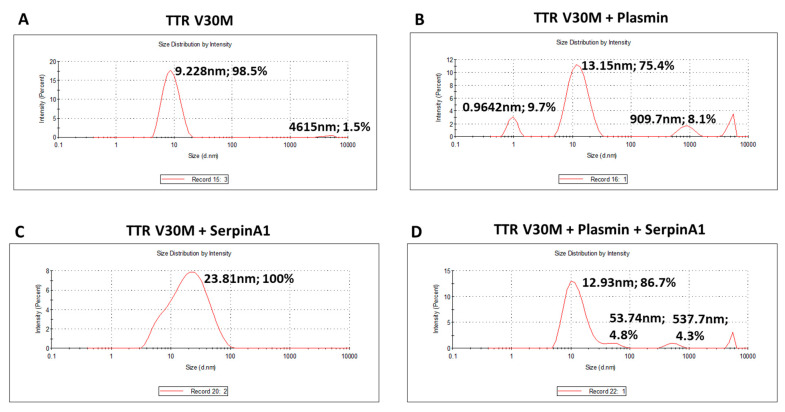
Plasmin enhances the transthyretin V30M aggregation, while SerpinA1 inhibits the process. Dynamic light scattering analysis was performed upon transthyretin V30M incubation at 37 °C for 24 h: (**A**) alone; (**B**) with plasmin; (**C**) with SerpinA1; (**D**) with plasmin and SerpinA1. The data results from three independent in vitro experiments, each one performed in triplicate.

The presence of TTR fragments, particularly the 49–127 C-terminal peptide, was implicated in TTR amyloidogenesis. Studies of in vitro TTR cleavage indicated that this fragment generated upon digestion with trypsin or plasmin was rapidly incorporated into amyloid fibrils, suggesting that TTR proteolysis facilitated the process of TTR aggregation [19,20,21,22]. Similarly, in the present work, we investigated the influence of plasmin-mediated proteolysis on the aggregation potential of TTR V30M. Upon 24 h of incubation, both plasmin-digested and non-digested samples were characterized using dynamic light scattering (DLS) analysis (Figure 2). Plasmin-mediated proteolysis facilitates the process of TTR aggregation, as observed by the increase of TTR aggregated species (909.7 nm; 8.1%) along with the decrease of the soluble form (13.15 nm; 75.4%) (Figure 2B), comparatively to non-digested samples, in which TTR was only found in the soluble form (9.228 nm; 98.5%) (Figure 2A). In addition, TTR V30M incubated with SerpinA1 revealed the presence of soluble particles exhibiting a large diameter (23.81 nm, 100%) (Figure 2C), probably indicating the formation of TTR-SerpinA1 complex, as described previously [33]. Samples incubated with both plasmin and SerpinA1 presented less abundant and smaller TTR aggregates (537.7 nm; 4.3%) (Figure 2D), as compared to samples only incubated with plasmin (Figure 2B).

**Figure 3:**
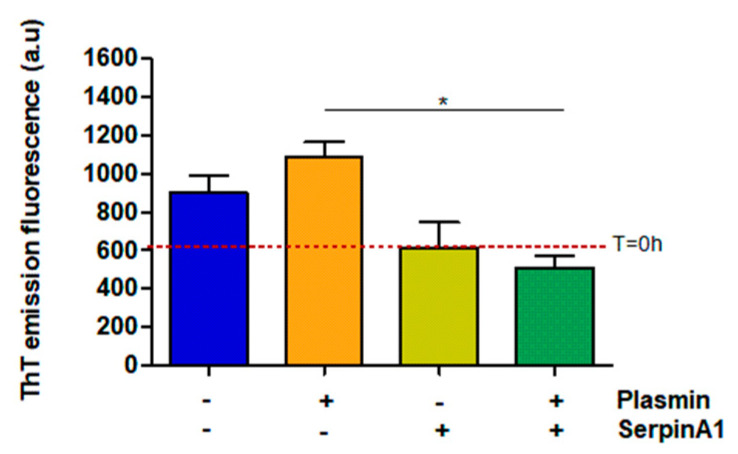
Transthyretin amyloid formation is favored upon plasmin-mediated proteolysis, being partially inhibited by SerpinA1. Thioflavin T experiments were performed upon transthyretin V30M incubation with plasmin and/or SerpinA1, at 37 °C for 24h. The fluorescence emission signal of thioflavin T of transthyretin V30M at the beginning of the experiment (T = 0 h) is represented as the red dotted line around 607. The data results from three independent in vitro experiments (*n* = 3). Statistical analysis was performed using one-way ANOVA with Tukey’s multiple comparison as post-test. * *p* < 0.05 in the presence of plasmin/presence of SerpinA1 vs. presence of plasmin/absence of SerpinA1, q = 6.092, df = 3. Effect size (r) = 0.924, odds ratio = 0.69 for an interval of confidence of 95%.

Besides its function as a serine protease inhibitor, SerpinA1 also functions as an extracellular chaperone and recently, it was reported that SerpinA1 inhibited TTR amyloid formation in vitro [33]. Thus, the same samples were analyzed by thioflavin T (ThT) assays to evaluate the amyloid nature of the formed species. The results demonstrated that plasmin facilitates TTR V30M amyloid formation, as observed by the increased ThT emission fluorescence signals upon plasmin incubation (1086 ± 75.68 vs. 901.3 ± 91.88 in the absence of plasmin) (Figure 3). Moreover, in the presence of plasmin, TTR V30M amyloid formation was significantly inhibited by SerpinA1 (510 ± 60.58 vs. 1086 ± 75.68 in the absence of SerpinA1; *p* < 0.05). SerpinA1 per se seemed to inhibit TTR V30M amyloid formation (613 ± 134 vs. 901.3 ± 91.88 in the absence of SerpinA1) (Figure 3).

### 2.3. SerpinA1 Downregulation Increased Transthyretin Deposition in the Heart of Old Transgenic Mice Carrying Human Transthyretin V30M Mutation

**Figure 4:**
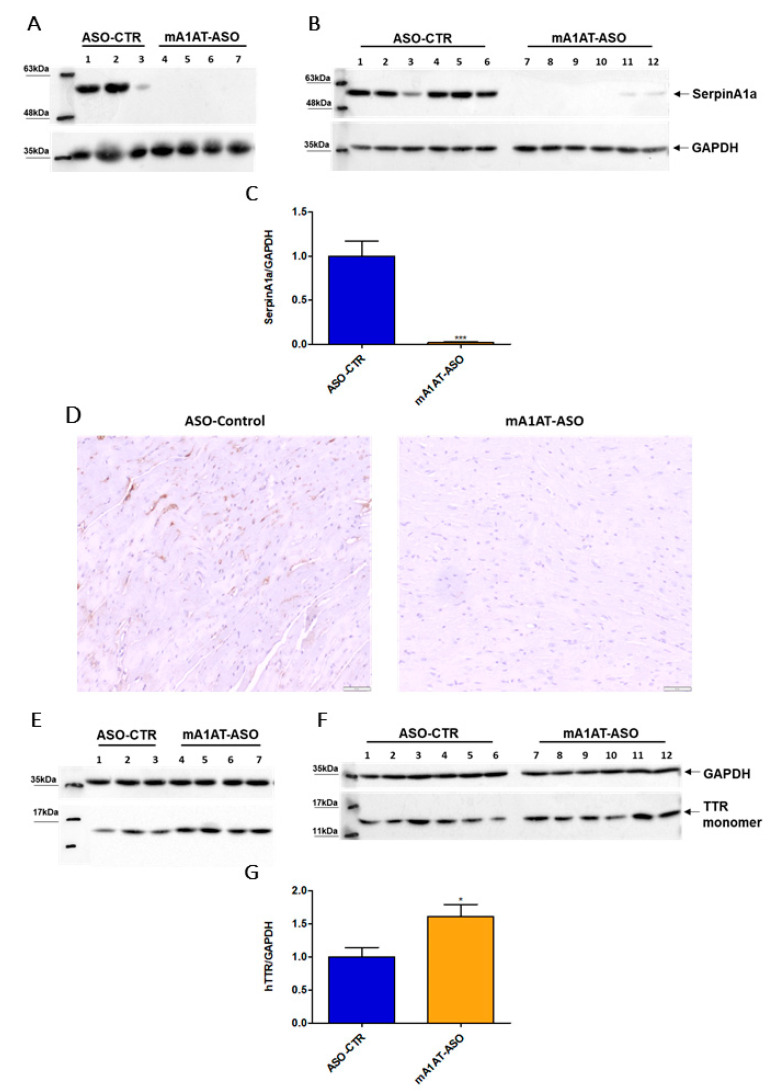
Human transthyretin was found to be increased in the heart of transgenic mice carrying human transthyretin V30M mutation upon SerpinA1 downregulation. Western blotting analysis of SerpinA1 and GAPDH expression in the heart of old (16–21 months, *n* = 7) (**A**) and young (12–13 months, *n* = 12) (**B**) mice. Quantification of SerpinA1 expression normalized to GAPDH (loading control protein) of the two pooled experiments (**C**). Immunohistochemistry data also reveal that SerpinA1 was effectively downregulated in the heart of HM30 mice. Images were captured at 10× magnification using Olympus BX50 microscope. Scale bar = 20 μm (**D**). Western blotting of TTR and GAPDH expression in mice cardiac tissue of both old (**E**) and young (**F**) animals. Bar plot represents the quantification of transthyretin expression normalized to GAPDH of the two pooled in vivo experiments (**G**). Protein bands were quantified by densitometry using Image J. Statistical analysis was performed using an unpaired *t*-test. *** *p* < 0.0001 when comparing the SerpinA1/GAPDH ratio between ASO-CTR and mA1AT-ASO, t = 6.026, df = 17; * *p* < 0.05 when comparing the hTTR/GAPDH ratio between ASO-CTR and mA1AT-ASO, t = 2.554, df = 17. Calculations were performed for an interval of confidence of 95%.

Previous studies reported that SerpinA1 knockdown was accompanied by an increase in TTR mRNA expression, as well as TTR protein levels in HepG2 cells. In collaboration with our group, it was also reported that SerpinA1 knockdown resulted in an increase in TTR mRNA expression in mouse liver, as well as in TTR protein levels in plasmas of transgenic mice carrying human TTR V30M mutation (HM30) [34]. Therefore, we decided to investigate whether SerpinA1 was also specifically downregulated in the mouse heart and whether effects on TTR protein levels could be observed. For that, SerpinA1-specific ASOs were subcutaneously administered to HM30 mice once a week for six weeks. Western blotting analysis (Figure 4A–C), as well as immunohistochemistry (Figure 4D), confirmed that SerpinA1 was effectively downregulated in the heart from younger and older animals (0.018 ± 0.01 vs. 1.0 ± 0.18 in ASO-CTR, *p* < 0.0001). In addition, a significant increase in TTR protein in mice cardiac tissue was observed (1.6 ± 0.18 vs. 1.0 ± 0.14 in ASO-CTR group, *p* = 0.020) (Figure 4E–G).

**Figure 5:**
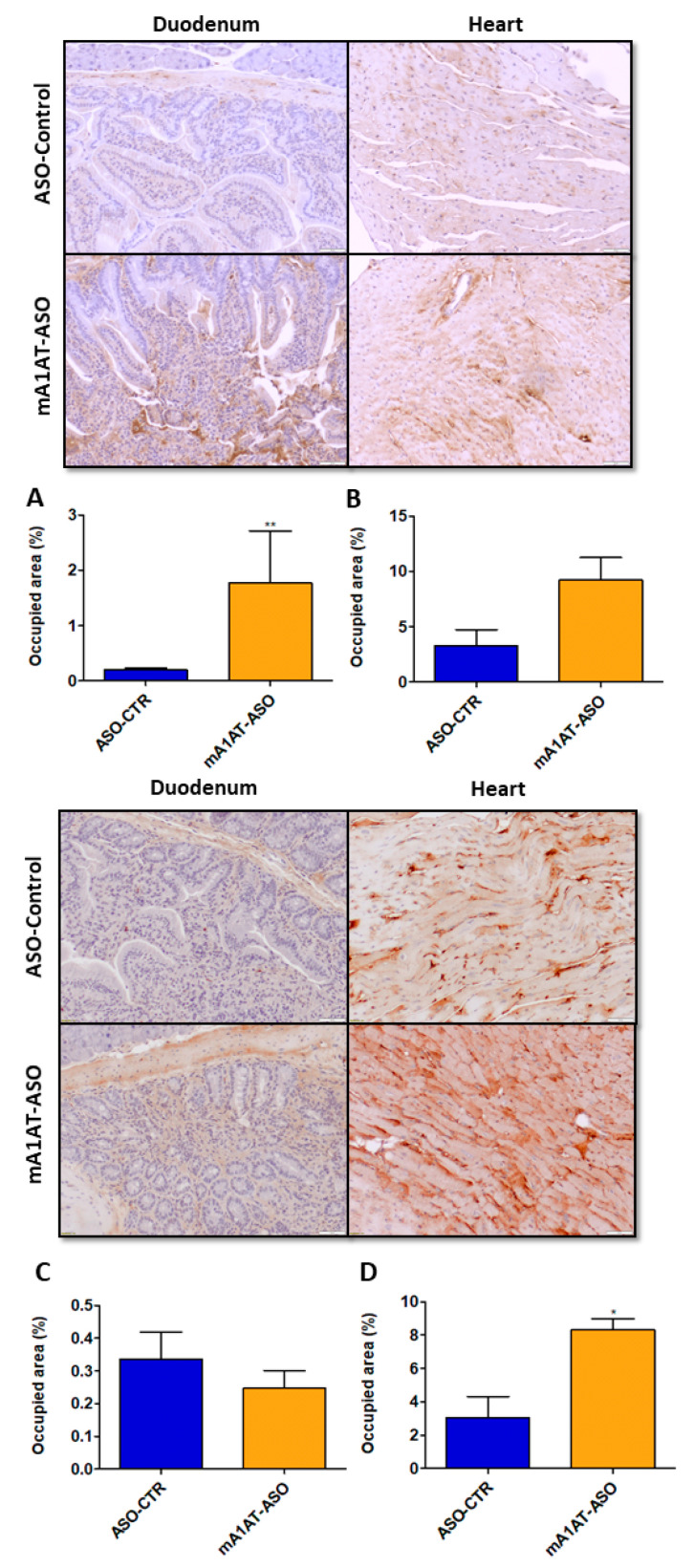
Transthyretin deposition is increased upon SerpinA1 knockdown in the mouse heart of older animals. Representative images of immunohistochemical analysis of mouse heart and duodenum upon the administration of antisense oligonucleotides targeting SerpinA1 to young (12–13 months, *n* = 12) (upper panel) and old (16–21 months, *n* = 7) (lower panel) animals. Bar plot representation of TTR quantification normalized to the total occupied area in the duodenum (**A**,**C**) and heart (**B**,**D**) of young (**A**,**B**) and old (**C**,**D**) mice. Images were captured at 10× magnification using Olympus BX50 microscope and analyzed using Image Pro Plus software. Scale bar = 20 μm. Statistical analysis was performed using an unpaired *t*-test. ** *p* < 0.01 comparing TTR deposition in the duodenum of younger animals in ASO-CTR vs. mA1AT-ASO, t = 3.300, df = 10; * *p* < 0.05 comparing TTR deposition in the heart of younger animals in ASO-CTR vs. mA1AT-ASO, t = 3.969, df = 5. Calculations were performed for an interval of confidence of 95%.

Our previous data revealed that, along with increased TTR mRNA and protein levels, SerpinA1 knockdown increased in vivo TTR deposition in several tissues, such as dorsal root ganglia (DRGs) and intestine [34]. In this study, we addressed whether TTR deposition was also promoted in mouse cardiac tissue after SerpinA1 downregulation. In younger animals, duodenal TTR deposition was found to be increased upon silencing of SerpinA1 (1.773 ± 0.94 vs. 0.1951 ± 0.04 in ASO-CTR, *p* = 0.0087) (Figure 5, upper panel), and a similar tendency was also observed in older animals (Figure 5, lower panel). Additionally, immunohistochemistry analysis demonstrated that TTR deposition was favored in the heart of older animals upon SerpinA1 downregulation (8.307 ± 0.6697 vs. 3.049 ± 1.269 in ASO-CTR, *p* = 0.0106) (Figure 5, lower panel), comparatively to the younger group (Figure 5, upper panel).

### 2.4. Transthyretin Fragments Are Observed in Mouse Cardiac Tissue upon SerpinA1 Downregulation

**Figure 6:**
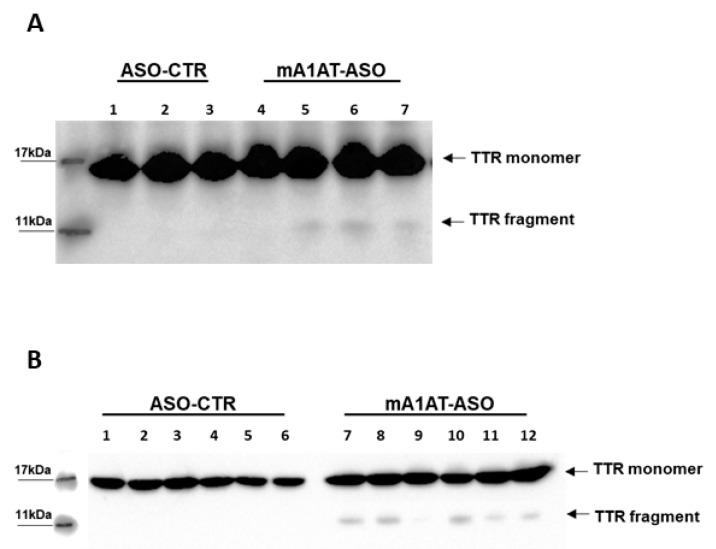
Western blotting analysis revealed the presence of transthyretin fragments upon SerpinA1 downregulation in the heart. Transthyretin fragments were detected in the heart of both older (**A**) and younger (**B**) animals.

Based on our experiments of in vitro plasmin-mediated proteolysis described above, indicating that SerpinA1 inhibits TTR cleavage, we evaluated the impact of SerpinA1 knockdown on TTR cleavage in vivo. Western blotting analysis revealed the presence of a protein band below to the monomeric TTR, corresponding to TTR fragment (<11 kDa) in mouse cardiac tissue in younger (Figure 6A) and older animals (Figure 6B). Similarly, these fragments were only detected using the antibody anti-TTR mutant (Y78F), clone AD7. In opposition, no TTR fragments were found upon SerpinA1 knockdown neither in other tissues of TTR deposition, such as duodenum (Supplementary data Figure S4A) and stomach (Supplementary data Figure S4B) nor in mice plasmas (Supplementary data Figure S4C).

### 2.5. SerpinA1 Downregulation Increases Proteolytic Activity, Particularly Plasmin Activity, in Plasmas of Transgenic Mice Carrying Human Transthyretin V30M Mutation

**Figure 7:**
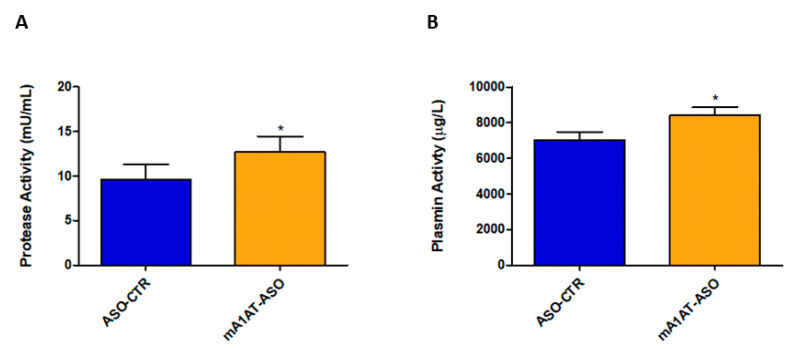
Serine protease activity and, particularly plasmin activity was found to be increased in mice plasmas upon SerpinA1 knockdown. Serine protease activity (**A**) and plasmin activity (**B**) were measured in plasma samples of HM30 mice according to the manufacturer’s instructions. Statistical analysis was performed using an unpaired *t*-test. * *p* < 0.05 comparing protease activity between ASO-CTR and mA1AT-ASO, t = 2.189, df = 14; * *p* < 0.05 comparing plasmin activity between ASO-CTR and mA1AT-ASO, t = 2.235, df = 16. Calculations were performed for an interval of confidence of 95%.

SerpinA1 partially inhibits plasmin-mediated proteolysis in vitro. Thus, fluorescence-based enzymatic assays were performed to evaluate the effects of downregulation of SerpinA1 on serine protease activity, namely plasmin activity in vivo. In fact, serine protease activity was effectively increased in plasmas of HM30 mice upon SerpinA1 downregulation (12.69 ± 1.746 vs. 9.606 ± 1.675 in ASO-CTR, *p* = 0.046) (Figure 7A) while no proteolytic activity was found in mice heart homogenates (Figure S5). In particular, the activity of plasmin was also found to be increased in mice plasmas after SerpinA1 knockdown (8422 ± 432 vs. 7013 ± 458 in ASO-CTR, *p* = 0.040) (Figure 7B).

## 3. Discussion

Tetramer destabilization is considered the rate-limiting step driving TTR amyloidogenesis. However, TTR proteolysis has been reported as an additional mechanism contributing to TTR amyloid formation [36]. The 49–127 C-terminal fragment is the most frequently encountered in ex vivo amyloid fibrils [13,15], and, the fragmentation pattern at Lys residues indicates the activity of a trypsin-like serine protease [18,37].

Bellotti and collaborators identified three TTR fragments upon in vitro incubation of the highly amyloidogenic TTR S52P with trypsin, being the 49–127 C-terminal fragment more prone to aggregation than the 16–127 and 81–127 C-terminal fragments [19]. A similar pattern of fragmentation was obtained with plasmin, a ubiquitous, widely distributed serine protease related to fibrinolysis [22].

Recently, the serine protease inhibitor, SerpinA1, was implicated in ATTR amyloidogenesis, and we hypothesized that it could also modulate TTR proteolysis. Previous studies by Niemietz et al. demonstrated that SerpinA1 inhibited TTR aggregation both in vitro, in cell culture experiments using hepatocyte-like cells (HLCs), and in vivo, in a study of SerpinA1 knockdown in mice carrying human TTR V30M mutation (HM30) [33,34].

In this study, our aim was to investigate the role of SerpinA1 as an inhibitor of serine proteases and its effect on the in vitro and in vivo modulation of TTR amyloid formation to contribute to a better knowledge of the process and to search for new and more specific therapeutic approaches.

In this sense, we confirmed that TTR V30M, the most frequent TTR variant related to ATTR amyloidosis, was prone to plasmin-mediated proteolysis in vitro and, that the cleaved protein aggregates more rapidly than the non-cleaved TTR V30M. N-terminal sequencing and MS analysis of the bands corresponding to TTR fragments generated by plasmin-mediated proteolysis identified the presence of the peptides 1–48 N-terminal and 49–127 C-terminal. The N-terminal region of the TTR polypeptide chain was enriched in hydrophobic amino acid residues and, importantly, the 26–57 TTR segment, belonging to the aggregation-prone regions (APR), exhibited high amyloid propensity [18]. These APR were protected when the protein was in its native form [38]. However, the destabilization of the native TTR structure induced by the single-point mutation at position 30 might expose those regions to cleavage, and, for that, the fragment 1–48 N-terminal TTR fragment may also be potentially considered highly amyloidogenic. It has been demonstrated that the 49–127 C-terminal fragment facilitates TTR amyloid formation in vitro [19,20,21,22], and, accordingly, Dasari et al. recently determined that the proteolytic cleavage of the K48-T49 peptide bond in the CD loop accelerated the formation of small spherical oligomers, which exhibited cytotoxic effects in neuroblastoma SH-SY5Y cells [39]. It was also shown that TTR aggregates generated by full-length or truncated TTR forms exhibited nearly identical molecular structural features, suggesting that TTR proteolysis in the CD loop destabilizes the native TTR tetramer. This destabilization of the TTR tetramer promotes oligomer formation through a similar mechanism of TTR misfolding and aggregation rather than through another molecular mechanism [39].

Marcoux et al. suggested the influence of biomechanical forces, particularly shear stress forces generated by fluid flow, on TTR proteolysis, which could influence the tissue specificity of TTR amyloid deposition. Indeed, a mechano-enzymatic cleavage mechanism for TTR proteolysis was proposed, where tetrameric TTR might be cleaved prior to TTR deposition and, then, due to strong shear stress observed in the heart, the C-terminal fragments would be released being rapidly incorporated into amyloid fibrils. Alternatively, both cleavage and dissociation may occur simultaneously at the heart, where both local shear stress forces and the relevant protease could be present [20]. These shear and interfacial forces are particularly strong in the cardiac tissue [40], which might explain the frequently encountered type-A fibrils in TTR deposits found in the heart. However, the presence of type-A amyloid fibrils in other tissues, such as the vitreous humor and the spinal cord of ATTR V30M patients, indicate that this mechanism would not explain the formation of TTR amyloid deposits based on their tissue-specific location, since these shear stress conditions were not observed neither in the eye nor in the central nervous system [41,42,43]. Moreover, in a recent study of Suhr et al., 14 out of 15 families with ATTR V30M amyloidosis exhibited a similar amyloid fibril composition within family members, independently of the age-onset disease. These observations indicate that, besides specific tissue/organ characteristics, genetic and/or epigenetic alterations may influence the amyloid fibril composition [44].

Our in vitro results using recombinant TTR show that SerpinA1 partially inhibits plasmin-mediated TTR proteolysis and suggest that, in parallel, it can also have an effect on the inhibition of TTR V30M amyloid formation seem to be independent of the presence of plasmin. Interestingly, this effect was compatible with the physical interaction between SerpinA1 and TTR that was recently suggested [33]. In addition, our DLS data indicate the presence of large diameter soluble particles, possibly the SerpinA1-TTR complex. Thus, future studies should be performed to clarify whether SerpinA1 performs an important role as a modulator of TTR proteolysis through its interaction with TTR, avoiding the access of plasmin to its targeting region in the TTR structure.

Our previous studies of SerpinA1 downregulation showed significantly increased TTR serum levels in HM30 mice, as well as in hepatoma cells [34]. Furthermore, SerpinA1 knockdown led to increased TTR deposition in the gastrointestinal tract, as well as in the sciatic nerve and dorsal root ganglia (DRG) of HM30 mice. In this work, we also found increased TTR protein deposits in the heart of older HM30 mice, whereas increased duodenal TTR deposition was found in the younger mice and, the same tendency was also observed in older ages. Moreover, we detected TTR fragments in mouse cardiac tissue upon SerpinA1 downregulation, while no TTR fragments were detected in mice plasmas nor in deposits from other tissues, such as the duodenum and stomach. The absence of fragments in mice plasmas upon SerpinA1 knockdown, as revealed in AD7 immunoblot, might be related to a very low concentration of TTR fragments in plasma and/or to insufficient sensitivity of the method. Additionally, we found increased serine protease activity, particularly plasmin activity, in plasmas upon treatment with ASOs targeting SerpinA1, whereas no proteolytic activity was observed in the heart of HM30 mice (Figure S3).

Despite the increasing interest in TTR proteolysis as a leading mechanism-driving TTR amyloidosis, some questions remain to be answered, namely whether TTR fragmentation occurs, prior to or after TTR aggregation/deposition and where it occurs. Some authors reported that plasmin degrades amorphous protein aggregates, releasing smaller soluble protein fragments, which were cytotoxic to both endothelial and microglial cells [45]. Trypsin or trypsin-like enzymes directly cleave acid-induced aggregates of full-length TTR V30M and barely cleave native soluble TTR V30M tetramer [46]. Additionally, recent cryo-electron microscopy (cryo-EM) experiments revealed the co-existence of both N-terminal and C-terminal TTR segments in one TTR fibril and, the relative special arrangement of these two segments are compatible with full-length TTR, suggesting that the process of fibril formation precedes TTR proteolysis [47]. In opposition, other studies showed increased proteolytic activity in plasmas from ATTR patients compared to healthy controls, suggesting that TTR proteolysis occurs in the bloodstream prior to TTR aggregation/deposition [31]. Accordingly, our data demonstrating increased protease and plasmin activity in mice plasmas, along with the absence of protease activity in mice hearts, suggest that in vivo TTR proteolysis occurs before fibril formation (Figure 7A,B, and supplementary data Figure S5).

In summary, our in vitro experiments demonstrate that plasmin cleaves the recombinant TTR V30M proteolytically and promotes its aggregation in vitro. Additionally, SerpinA1 partially inhibits the activity of plasmin in vitro, which decreases TTR amyloid formation. To investigate the relevance of these findings in vivo, SerpinA1 expression was knockdown in HM30 mice. The absence of SerpinA1 favored TTR deposition in mice tissues and increased the serine protease activity, namely plasmin activity in mice plasmas, which was accompanied by the presence of TTR fragments in the mice heart.

This work presents some limitations in particular concerning the knowledge of the detailed mechanism involving SerpinA1 inhibition of plasmin-mediated TTR proteolysis and also the impact of SerpinA1 downregulation in mice carrying TTR WT or carrying non-V30M TTR mutations. Accordingly, future experiments must be performed namely to dissect the molecular mechanisms by which SerpinA1 inhibits plasmin activity through direct interaction with the protease or by the formation of TTR-SerpinA1 complex. Additionally, it would also be interesting to evaluate the role of plasmin on TTR proteolysis using different animal models developing ATTR amyloidosis, such as transgenic mice carrying human A97S mutation [48]. Ultimately, it is important, to investigate plasmin and other extracellular serine proteases activity in TTR V30M patients and patients carrying other TTR amyloidogenic mutations to evaluate whether this activity has tissue-specific effects or is related to disease progression potentiating its interest as a biomarker in ATTR amyloidosis.

Altogether our in vitro and in vivo results show that plasmin is a plausible protease performing a role on TTR proteolysis and reveal SerpinA1 as an important modulator of the process of TTR cleavage. Our findings might contribute to the development of more effective and targeted therapies for the treatment of ATTR amyloidosis.

## 4. Materials and Methods

### 4.1. Reagents

Native human plasmin protein (active), native human SerpinA1 protein (active), protease activity assay kit, plasmin activity assay kit, and recombinant rabbit anti-GAPDH antibody were purchased from Abcam (Cambridge, UK). Mouse monoclonal antibody anti-TTR mutant (Y78F), clone AD7 was from Merck Millipore (Sigma-Merck, Darmstadt, Germany). Rabbit polyclonal anti-human TTR antibody was from DAKO (Hovedstaden, Denmark). Rat monoclonal anti-mouse SerpinA1 antibody was from R&D systems (Minneapolis, MN, USA). Pierce TM High Capacity Endotoxin Removal Resin was from Thermo Scientific (Waltham, MA, USA). GalNAc-AAT ASO (mA1AT-ASO: ACCCAATTCAGAAGGAAGGA) and GalNAc-Control ASO (ASO-CTR: CCTTCCCTGAAGGTTCCTCC) [48] were kindly provided by Ionis Pharmaceuticals (Carlsbad, CA, USA).

### 4.2. Recombinant Human Transthyretin

TTR V30M and TTR WT were produced using a bacterial expression system and purified as previously described [49]. Recombinant TTR was applied to an affinity chromatography column packed with Pierce TM High Capacity Endotoxin Removal to remove bacterial lipopolysaccharides according to the manufacturer’s instructions. TTR was then dialyzed against endotoxin-free phosphate-buffered saline (PBS) (Sigma-Merck, Darmstadt, Germany), concentrated using Vivaspin ultrafiltration units (GE Healthcare, Chicago, IL, USA), and quantified using Bradford protein assay (Bio-Rad, Hercules, CA, USA).

### 4.3. In Vitro Plasmin-Mediated Proteolysis Assays

TTR variants were firstly filtered through a sterile 0.2 μm inorganic membrane ANOTOP syringe filter (Whatman, Maidstone, UK) to remove any protein aggregates. Then, TTR (18 μM) was incubated with plasmin (0.4U) and/or SerpinA1 (2.8 μM) at 37 °C for 24 h, under stagnant conditions. TTR proteolysis was stopped by using phenylmethylsulfonyl fluoride (PMSF) at a final concentration of 1.5 mM and, then TTR samples (500 ng/well) were applied into a 15% polyacrylamide SDS-PAGE. After electrophoresis, proteins were stained with PageBlue™ protein staining solution (Thermo Scientific, Waltham, MA, USA) or transferred onto nitrocellulose membrane using iBlot dry blotting system (Thermo Scientific, Waltham, MA, USA). TTR immunoblot was performed using a commercially available antibody produced in our lab, mouse anti-transthyretin mutant (Y78F), clone AD7 (1:100) (Sigma-Merck, Darmstadt, Germany). This monoclonal antibody detects glycosylated form of TTR V30M in plasma and acts as a conformational antibody recognizing specific TTR variants, such as G47A, G49A, S50R, and T59K, in particular conditions [28]. Rabbit polyclonal anti-human TTR (1:1000) (DAKO, Hovedstaden, Denmark) was also used. ECL chemiluminescent reagent (Bio-Rad, Hercules, CA, USA) was used as a detection method using Chemidoc apparatus (Bio-Rad, Hercules, CA, USA). Three independent experiments of in vitro plasmin-mediated proteolysis were performed.

### 4.4. N-Terminal Sequencing Analysis of TTR Fragments

Plasmin-digested TTR V30M samples (15 μg) were loaded into a 15% polyacrylamide SDS-PAGE gel. Samples were then transferred onto a PVDF membrane (Bio-Rad, Hercules, CA, USA) and proteins were further stained with Coomassie blue R-250 (VWR International, Radnor, PA, USA). Membranes were allowed to dry and the bands below to the TTR monomer, corresponding to TTR fragments (band 1 and band 2) were excised for N-terminal sequencing analysis (Edman degradation method) using an ABI Procise Protein Sequencer, an ABI Microgradient Pump System, and an ABI Programmable Absorbance Detector (Applied Biosystems Inc., Waltham, MA, USA).

### 4.5. Mass Spectrometry Analysis for the Identification of TTR Fragments

Gel bands excised from SDS-PAGE were washed twice with 50% acetonitrile (ACN) in 50 mM triethylammonium bicarbonate (TEAB) with shaking at 1500 rpm for 5 min and further treated with ACN twice. Then, proteins were reduced with 25 mM dithiothreitol (DTT) for 20 min at 56 °C and alkylated with 55 mM iodoacetamide (IAA) for 20 min at room temperature in the dark, followed by the same wash procedure. Proteins were then digested with trypsin (240 ng) in 50 mM TEAB/0.01% surfactant (ProteaseMAX, Promega, Madison, WI, USA) for 60 min at 50 °C. Peptide gel extraction was performed with 2.5% trifluoroacetic acid (TFA) followed by 50% ACN, 0.1% TFA. Samples were dried using Speedvac, resuspended in 10mL 0.1% TFA and cleaned by C18 reverse phase chromatography according to manufacturer’s instructions (ZipTip, Sigma-Merck, Darmstadt, Germany).

Sample protein identification and quantification were performed by nano-liquid chromatography mass spectrometry (nano LC-MS/MS), as previously described [50] with a 90 min chromatographic separation run. This equipment was composed of an Ultimate 3000 liquid chromatography system coupled to a Q-Exactive Hybrid Quadrupole-Orbitrap mass spectrometer (Thermo Scientific, Bremen, Germany). A total of 500 nanograms of each TTR peptide were loaded onto a trapping cartridge (Acclaim PepMap C18 100 Å, 5 mm × 300 µm i.d., 160454, Thermo Scientific, Bremen, Germany) in a mobile phase of 2% ACN, 0.1% FA at 10 µL/min. After 3 min loading, the trap column was switched in-line to a 50 cm × 75 μm inner diameter EASY-Spray column (ES803, PepMap RSLC, C18, 2 μm, Thermo Scientific, Bremen, Germany) at 250 nL/min. The LC separation was achieved by mixing A: 0.1% FA and B: 80% ACN, 0.1% FA with the following gradient: 2 min (2.5% B to 10% B), 50 min (10% B to 35% B), 8 min (35% B to 99% B), and 10 min (hold 99% B). Subsequently, the column was equilibrated with 2.5% B for 17 min. The specific MS parameters were: MS maximum injection time, 100 ms; dd settings: minimum AGC target 7 × 103, intensity threshold 6.4 × 104, and dynamic exclusion 20 s. Data acquisition was controlled by Tune 2.11 software (Thermo Scientific, Bremen, Germany). The UniProt database 2020_05 for the Homo sapiens proteome (75069 entries) together with a customized TTR amino acid sequence were considered for protein identification. Protein identification was performed with the Proteome Discoverer software v2.5 (Thermo Scientific, Bremen, Germany). Proteins were quantified by Label-Free Quantification—LFQ, with precursor quantification based on intensity.

### 4.6. Aggregation Studies: Dynamic Light Scattering and Thioflavin T Assay

DLS measurements were performed at 25 °C using Malvern Zetasizer Nano ZS apparatus (Malvern, Worcestershire, UK). Each sample was measured 3 times, and the values exhibited in the curves were the average distributions from those triplicates. Thioflavin T (ThT) assay was performed in PBS (pH = 7.4) using a 96-well black bottom plate. TTR (12.5 μg) and ThT (30 μM) (Sigma-Merck, Darmstadt, Germany) per well were mixed and, the fluorescence was measured at Exc./Em = 450 nm/482 nm using SynergyMx apparatus (BioTeK, Winooski, VT, USA).

### 4.7. Mice

Mice were kept in a controlled temperature room and maintained under a 12 h light/dark period. Transgenic mice carrying human TTR V30M (HM30) were bred as described before [51]. Both younger (12–13 months; *n* = 12) and older (16–21 months; *n* = 7) HM30 mice were subcutaneously (s.c) injected once a week with 5 mg ASO/kg body weight following previous protocols [48]. Animals were euthanized at week 6 of treatment. Plasma and tissue sections were collected and frozen at −80 °C or fixed in formalin for further analysis.

### 4.8. Determination of SerpinA1 and TTR Protein Levels in Mice Heart

Mouse hearts were homogenized using RIPA lysis and extraction buffer according to the manufacturer’s instructions (Santa Cruz Biotechnology, Dallas, TX, USA). Briefly, mouse hearts were homogenized in RIPA buffer using a laboratory homogenizer to disrupt the tissue. Protein homogenates were further frizzed at −80 °C to promote cell lysis, further centrifuged at 21,500× *g* for 15 min at 4 °C and, the supernatant containing protein was harvested. Protein was quantified in the heart lysates using Bradford protein assay (BioRad, Hercules, CA, USA), and 50 μg of total protein was loaded into a 10% and 15% polyacrylamide SDS-PAGE to evaluate SerpinA1 and TTR expression, respectively. Gels were then transferred onto nitrocellulose membrane using a wet system and, membranes were incubated overnight with rat monoclonal anti-mouse SerpinA1 antibody (1:1000; R&D systems, Minneapolis, MN, USA), rabbit polyclonal anti-human TTR antibody (1:1000, DAKO) or mouse anti-transthyretin mutant (Y78F), clone AD7 (1:100, Sigma-Merck, Darmstadt, Germany). GAPDH was used as a protein loading control, and recombinant rabbit anti-GAPDH antibody was used for immunoblotting (1:100000, Abcam, Cambridge, UK). ECL chemiluminescence reagent (Bio-Rad, Hercules, CA, USA) was used as a detection method using Chemidoc apparatus (Bio-Rad, Hercules, CA, USA). Protein bands were quantified by densitometry using ImageJ (U. S. National Institutes of Health, Bethesda, MD, USA), and results of protein expression were normalized to GAPDH expression.

### 4.9. Immunohistochemical Analysis of Tissue TTR Deposition

Paraffin-embedded sections of both duodenal and cardiac tissue were deparaffinized in xylene and rehydrated in descent alcohol series. Antigen retrieval was performed at 95 °C for 15 min using citrate buffer (pH = 6) and, then, endogenous peroxidase activity was quenched in 3% hydrogen peroxide in methanol. Sections were blocked using 10% fetal bovine serum, 1% bovine serum albumin, and 0.5% Triton X-100 in PBS. TTR immunostaining was performed using primary rabbit polyclonal anti-human TTR antibody (1:600, DAKO, Hovedstaden, Denmark) and secondary anti-rabbit antibody (1:200) (Vector, Burlingame, CA, USA). For SerpinA1 staining, primary rat anti-mouse antibody (1:100; R&D systems, Minneapolis, MN, USA) and secondary anti-rat antibody (1:200) (Vector, Burlingame, CA, USA) were used. Tissue slides were developed using 3,3′-diaminobenzidine (DAB) (DAKO, Hovedstaden, Denmark), counterstained with hematoxylin and mounted in Entellan^®^ (Sigma-Merck, Darmstadt, Germany). Images were captured at 10x magnification using Olympus BX50 microscope (Shinjuku, Tokyo, Japan) and analyzed using Image Pro Plus software (Rockville, MD, USA). Results represent the occupied area in pixels corresponding to the substrate reaction color that was further normalized relative to the total image area.

### 4.10. Protease Activity and Plasmin Activity Fluorescence Measurements

Plasma samples and heart homogenates from HM30 mice were directly used without any dilution. However, protein from heart homogenates was extracted by using RIPA lysis and extraction buffer without supplementation with protease inhibitors. Protease activity assay and plasmin activity assay kits were used according to the manufacturer’s instructions. Briefly, mice samples were incubated with FITC-casein substrate in protease activity assay, whereas in the plasmin activity assay, samples were incubated with a synthetic plasmin AMC-substrate. The fluorescence was measured at Ex/Em = 485/530 nm and Ex/Em = 360/450 nm in protease activity assay kit (Abcam, Cambridge, UK) and plasmin activity assay kit (Abcam, Cambridge, UK), respectively, using SynergyMx apparatus (BioTek, Winooski, VT, USA). Both protease and plasmin activities were calculated according to the manufacturer’s instructions.

### 4.11. Statistical Analysis

Statistical analysis was performed by one-way ANOVA (Tukey’s multiple comparisons as post-test) and unpaired t-test using GraphPad Prism 5 software (San Diego, CA, USA). Statistical significance was considered when *p*-value ≤ 0.05. Results were expressed as mean + standard error of the mean (SEM).

## Figures and Tables

**Table 1:** Identification of transthyretin peptides upon plasmin digestion by N-terminal sequencing. Band 1 starts at the amino acid residue 49, whereas band 2 starts at the first amino acid residue in the transthyretin polypeptide chain, indicating that band 1 corresponds to the 49–127 C-terminal fragment and band 2 to the 1–48 N-terminal fragment.

TTR Fragment	Residue Number				
	1	2	3	4	5
#1	T	S	E	S	G
#2	G	P	T	G	T

## Data Availability

Data are contained within the article or Supplementary Materials.

## Supplementary Materials

Supplementary Materials can be found at www.mdpi.com/article/10.3390/ijms22179488/s1.
