# Supplementary material for: In Vitro and In Vivo Effects of SerpinA1 on the Modulation of Transthyretin Proteolysis
Source: Int J Mol Sci. 2021 Aug 31;22(17):9488. doi: 10.3390/ijms22179488 (PMC8430710; doi:10.3390/ijms22179488)
Supplement: Supplementary file 1 [file ijms-22-09488-s001.zip › Captions of Supplementary Materials.pdf]

Supplementary Materials

In Vitro and In Vivo Effects of SerpinA1 on the Modulation of Transthyretin Proteolysis

Filipa Bezerra [1,2], Christoph Niemietz [3], Hartmut H. J. Schmidt [3,†], Andree Zibert [3], Shuling Guo [4], Brett P. Monia [4], Paula Gonçalves [1], Maria João Saraiva [1,2] and Maria Rosário Almeida [1,2,*]

[1] Molecular Neurobiology Group, i3S-Instituto de Investigação e Inovação em Saúde, IBMC-Instituto de Biologia Molecular e Celular, Universidade do Porto, 4200-135 Porto, Portugal; carla.bezerra@ibmc.up.pt (F.B.); b12458@med.uminho.pt (P.G.); mjsaraiv@ibmc.up.pt (M.J.S.)

[2] Departamento de Biologia Molecular, ICBAS-Instituto de Ciências Biomédicas Abel Salazar, Universidade do Porto, 4050-313 Porto, Portugal

[3] Medizinische Klinik B, Universitätsklinikum Münster, 48149 Münster, Germany; Christoph.Niemietz@taconic.com (C.N.); hepar@ume.de (H.H.J.S.); Andree.Zibert@ukmuenster.de (A.Z.)

[4] Ionis Pharmaceuticals, Carlsbad, CA 92010, USA; sguo@ionisph.com (S.G.); bmonia@ionisph.com (B.P.M.)

* Correspondence: ralmeida@ibmc.up.pt

† Present address: Universitätsklinikum Essen, Germany.

Supplementary Materials: The following are available online at www.mdpi.com/xxx/s1

Table S1: TTR peptides obtained upon tryptic digestion of the TTR V30M fragments excised from SDS-PAGE gels**.**

Figure S1: Results of three independent experiments of *in vitro* plasmin-mediated proteolysis of TTR V30M in the presence or absence of SerpinA1

Figure S2: TTR WT was not cleaved by plasmin *in vitro* neither at 24h nor 48h. Western blotting analysis of the two independent *in vitro* experiments. Western blot was performed using mouse anti-TTR mutant (Y78F), clone AD7. Both the dimeric and the monomeric TTR forms were detected.

Figure S3A: First DLS experiment for determination of the aggregation potential of TTR V30M upon 24h of incubation with plasmin and/or SerpinA1. Results represent the average of three replicates *per* condition.

Figure S3B: Second DLS experiment for determination of the aggregation potential of TTR V30M upon 24h of incubation with plasmin and/or SerpinA1. Results represent the average of three replicates *per* condition.

Figure S3C: Third DLS experiment for determination of the aggregation potential of TTR V30M upon 24h of incubation with plasmin and/or SerpinA1. Results represent the average of three replicates *per* condition.

Figure S4: No TTR fragments were detected neither in other organs of TTR deposition nor in plasmas from HM30 mice. TTR fragments were not detected neither in the duodenum (A) nor stomach (B) of both older (16-21 months) (left) and younger (12-13 months) (right) animals. Additionally, no TTR fragments were encountered in mice plasmas (C). Western blot was performed using mouse anti-TTR mutant (Y78F), clone AD7.

Figure S5: No serine protease activity was found in the heart of HM30. Serine protease activity was measured in heart homogenates (without using protease inhibitors) of HM30 mice according to manufacturer's instructions. Heart homogenates were obtained. Background control (green line) corresponds only to assay buffer without any sample. No serine protease activity was detected in the cardiac tissue of both ASO-CTR (orange line) and mA1AT-ASO (blue line) mice groups.
