# Supplementary figures and images for: In Vitro and In Vivo Effects of SerpinA1 on the Modulation of Transthyretin Proteolysis
Source: Int J Mol Sci. 2021 Aug 31;22(17):9488. doi: 10.3390/ijms22179488 (PMC8430710; doi:10.3390/ijms22179488)

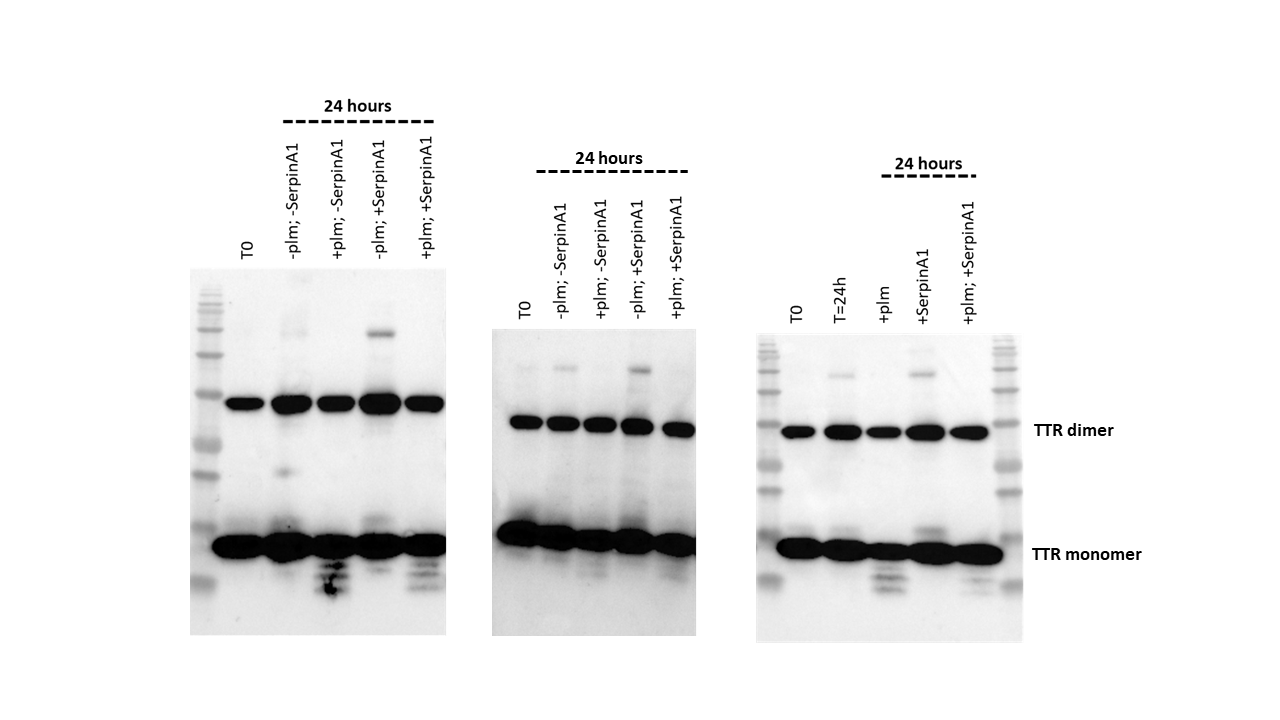

Supplement: Supplementary file 1 [file ijms-22-09488-s001.zip › Figure S1.tif]

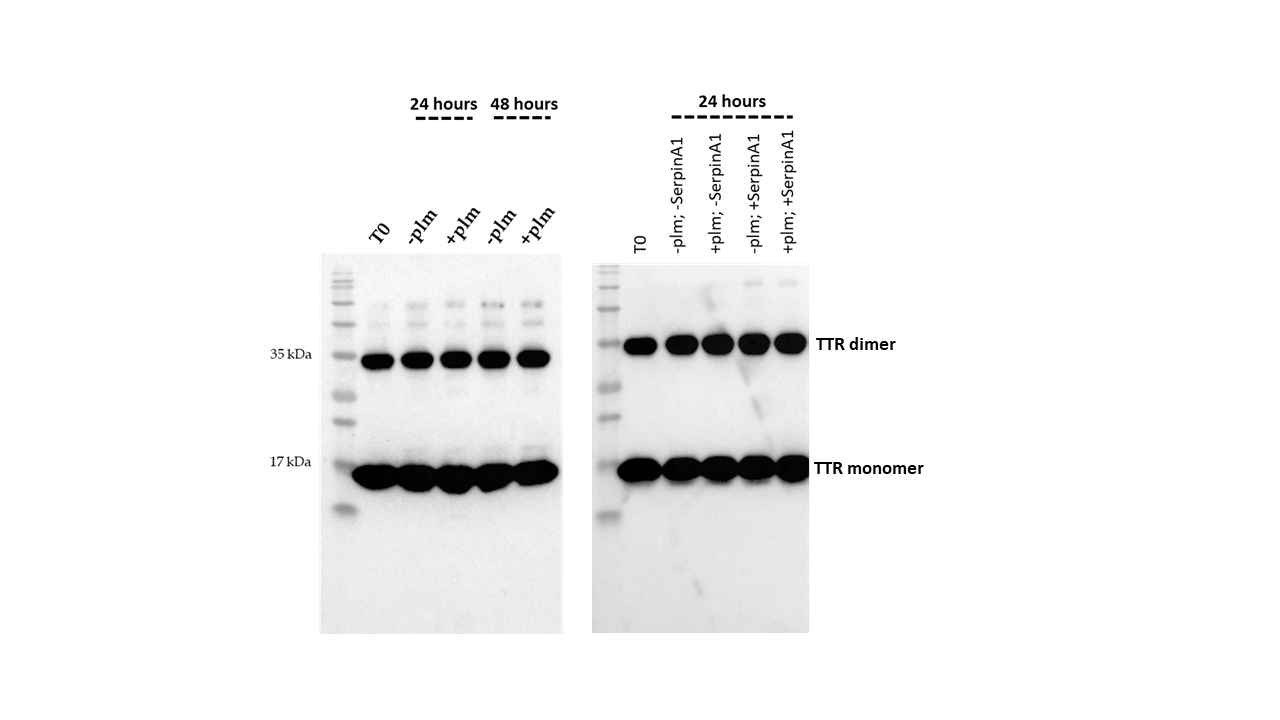

Supplement: Supplementary file 1 [file ijms-22-09488-s001.zip › Figure S2.tif]

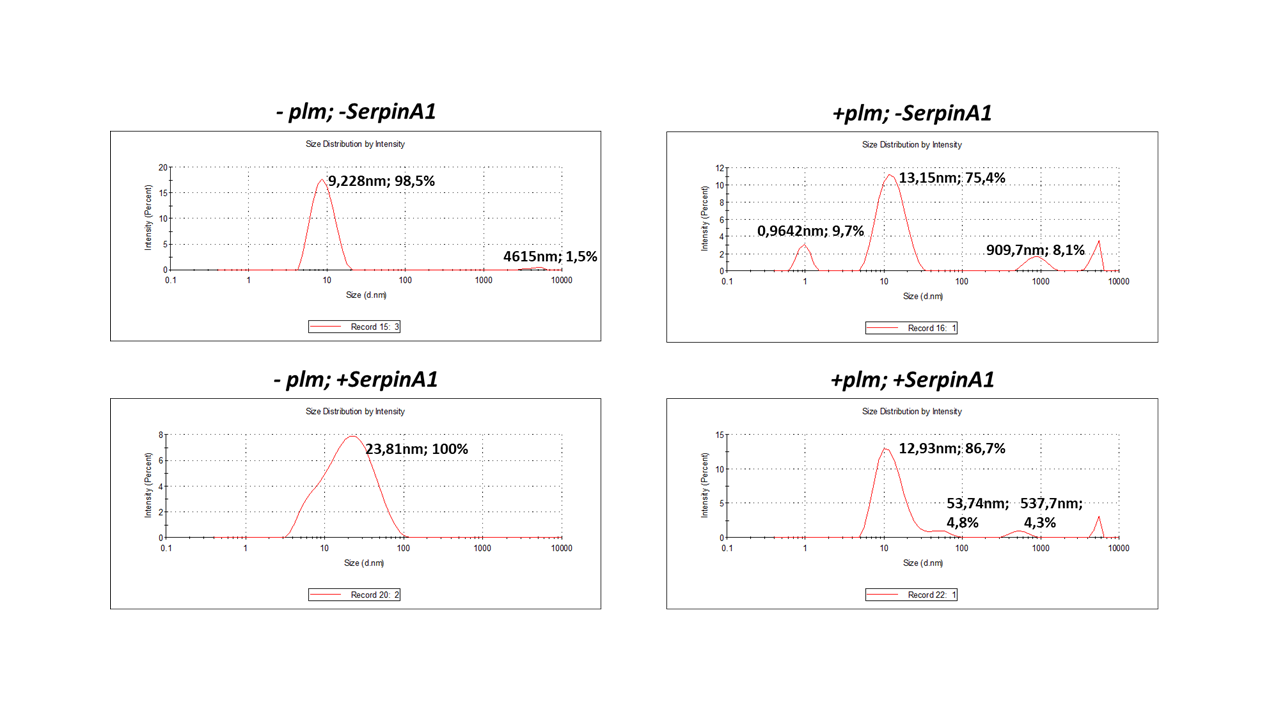

Supplement: Supplementary file 1 [file ijms-22-09488-s001.zip › Figure S3A.tif]

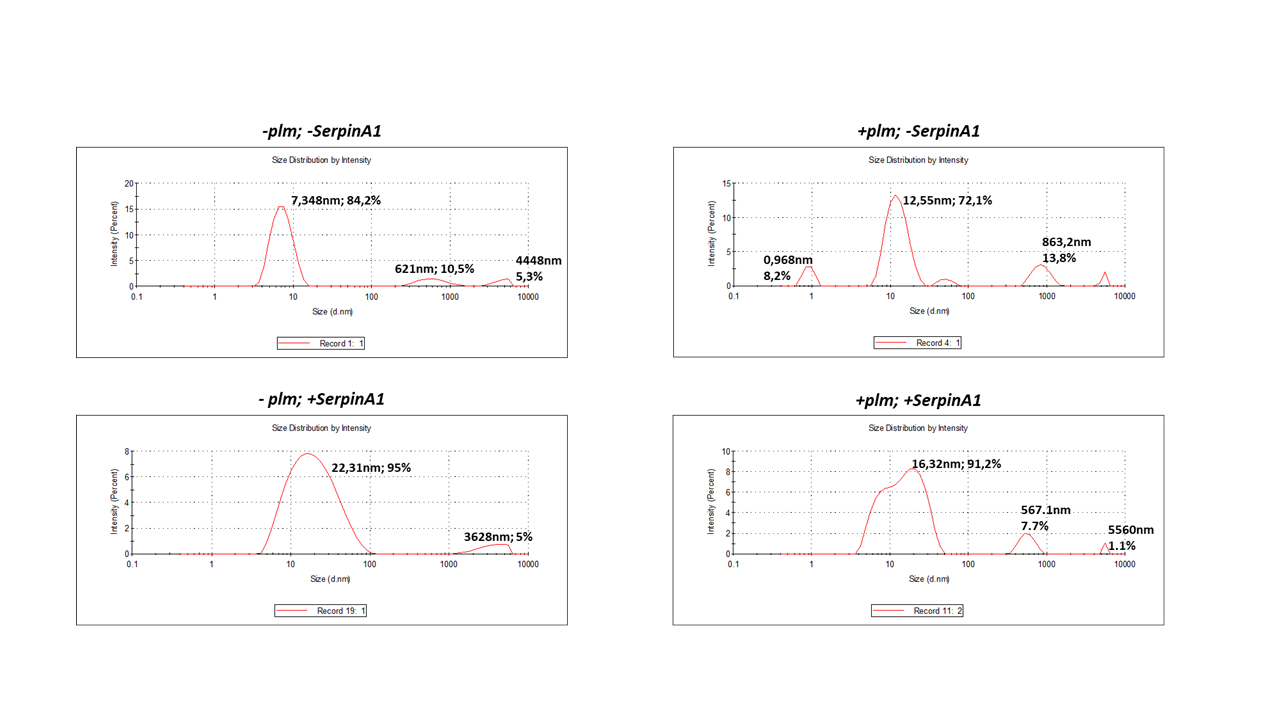

Supplement: Supplementary file 1 [file ijms-22-09488-s001.zip › Figure S3B.tif]

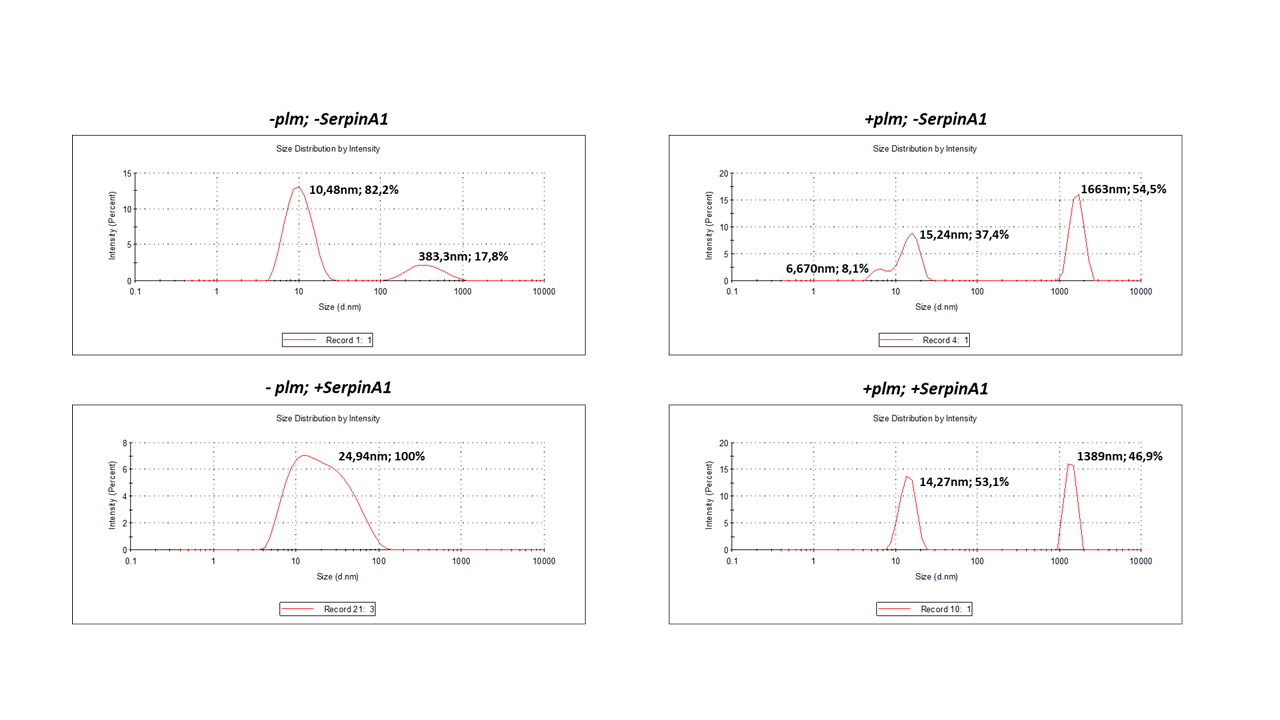

Supplement: Supplementary file 1 [file ijms-22-09488-s001.zip › Figure S3C.tif]

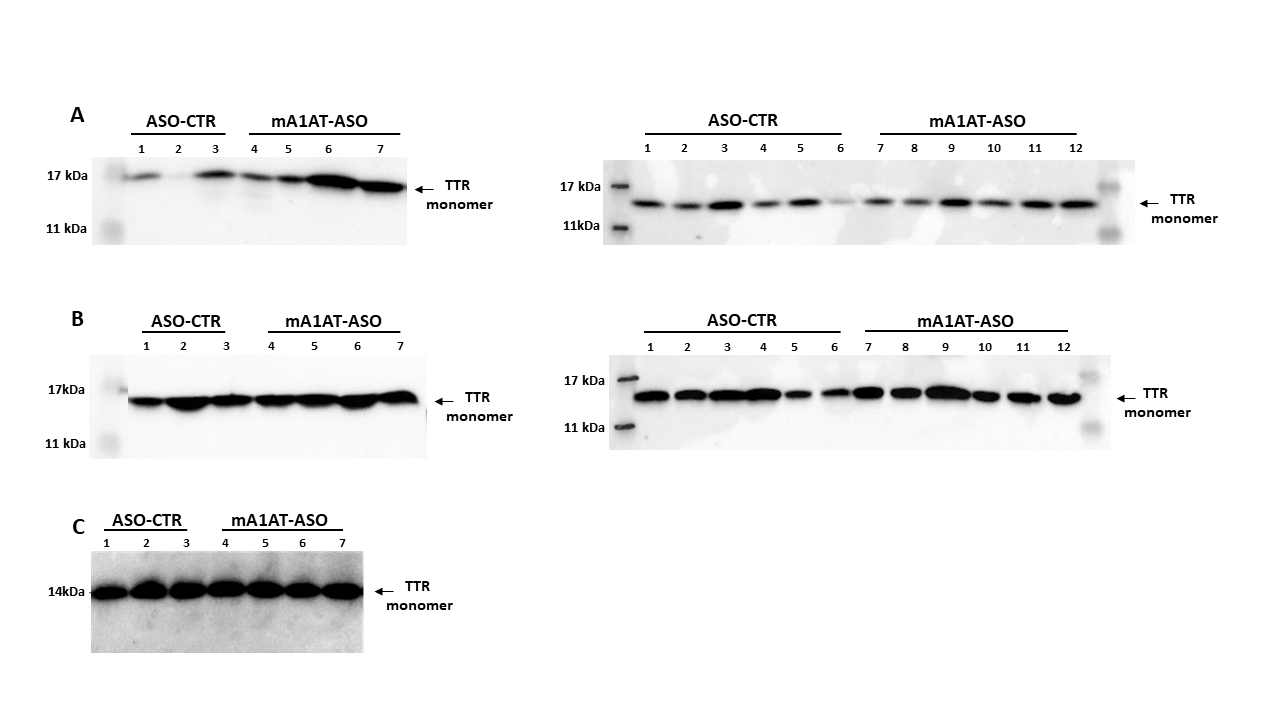

Supplement: Supplementary file 1 [file ijms-22-09488-s001.zip › Figure S4.tif]

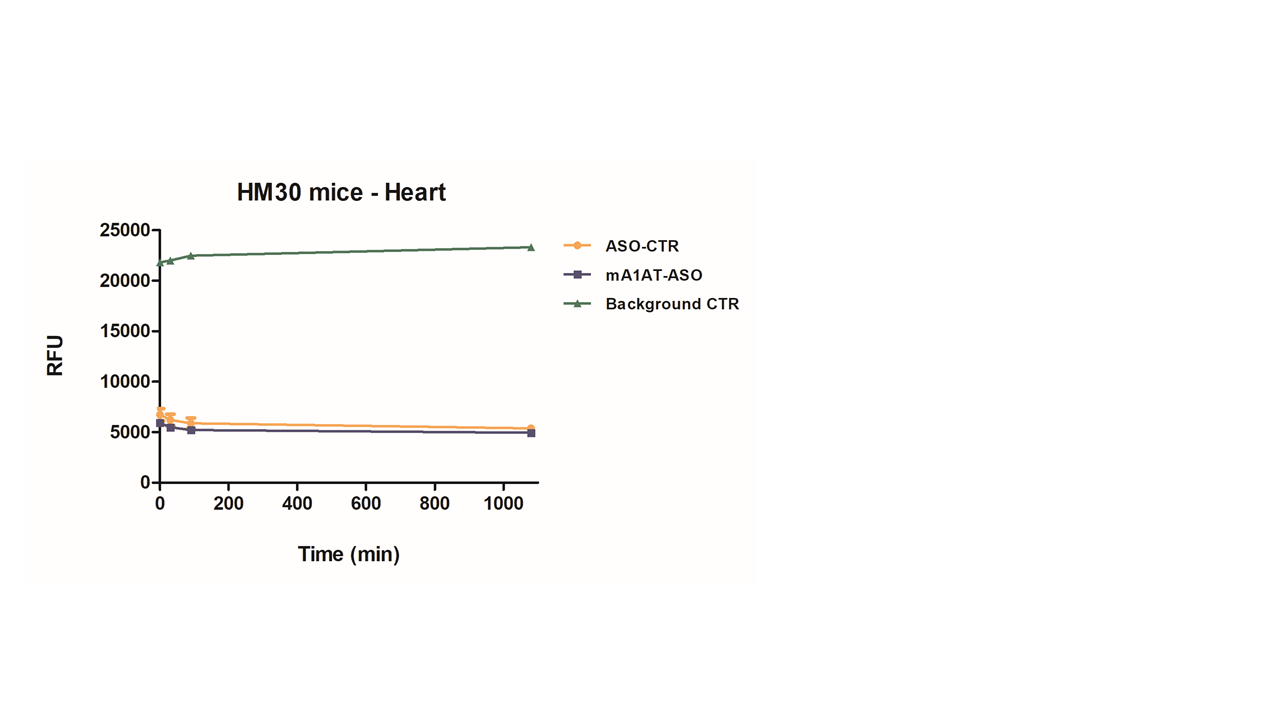

Supplement: Supplementary file 1 [file ijms-22-09488-s001.zip › Figure S5.tif]

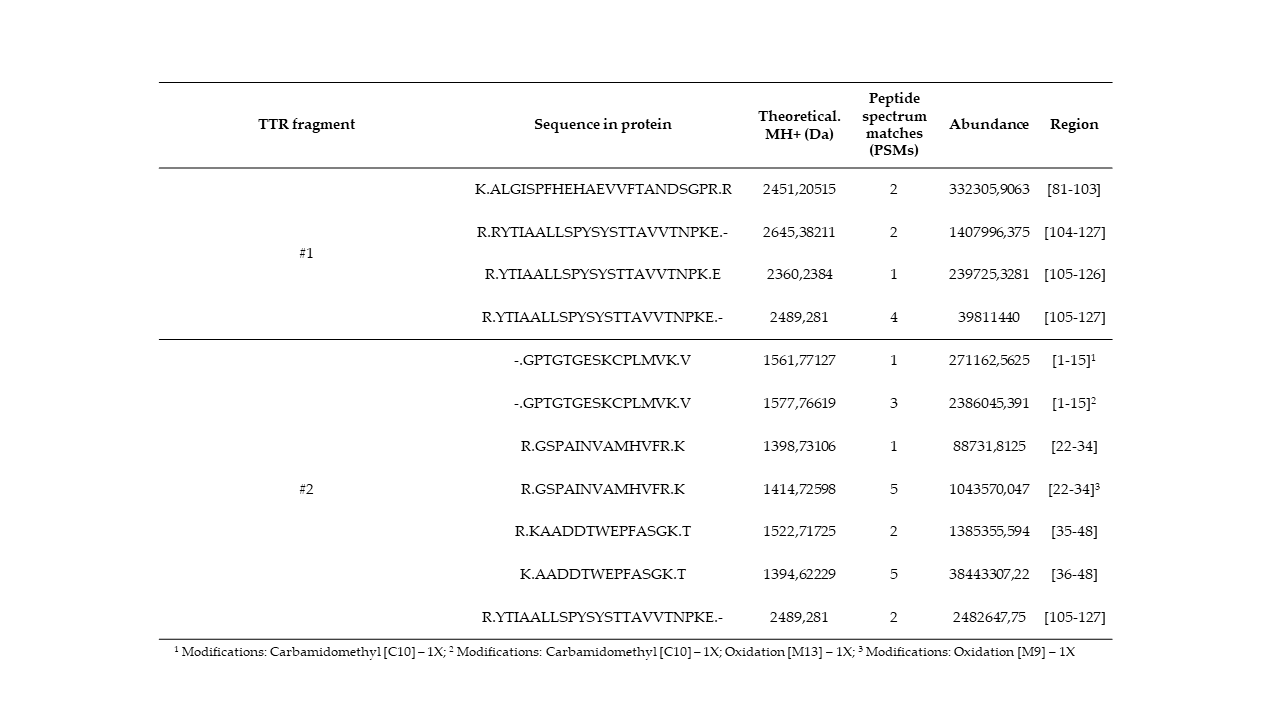

Supplement: Supplementary file 1 [file ijms-22-09488-s001.zip › Table S1.tif]
